# *Bacillus* coagulans and *Clostridium* butyricum synergistically alleviate depression in a chronic unpredictable mild stress mouse model through altering gut microbiota and prefrontal cortex gene expression

**DOI:** 10.3389/fphar.2024.1393874

**Published:** 2024-05-23

**Authors:** Jingyi Xu, Lei Zhou, Zhaowei Chen, Yuezhu Wang, Fang Xu, Qun Kuang, Yixuan Zhang, Huajun Zheng

**Affiliations:** ^1^ School of Life Science and Biopharmaceutics, Shenyang Pharmaceutical University, Shenyang, China; ^2^ Shanghai-MOST Key Laboratory of Health and Disease Genomics, NHC Key Lab of Reproduction Regulation, Shanghai Institute for Biomedical and Pharmaceutical Technologies, Shanghai, China; ^3^ The Academician Workstation, Shanghai Fourth People’s Hospital Affiliated to Tongji University, Shanghai, China; ^4^ Jiangsu Limited Company of Suwei Microbiology, Wuxi, China

**Keywords:** depression, chronic unpredictable mild stress, CUMS, gut microbiota, probiotics, *Bacillus* coagulans, *Clostridium* butyricum, transcriptome

## Abstract

**Introduction:** The prevalence of major depressive disorder (MDD) has gradually increased and has attracted widespread attention. The aim of this study was to investigate the effect of a probiotic compound consisting of *Bacillus coagulans* and *Clostridium butyricum*, on a mouse depression model.

**Methods:** Mice were subjected to chronic unpredictable mild stress (CUMS) and then treated with the probiotics at different concentrations. And mice received behavior test such as forced swimming test and tail suspension test. After that, all mice were sacrificed and the samples were collected for analysis. Moreover, prefrontal cortex (PFC) gene expression and the gut microbiota among different groups were also analyzed.

**Results:** Probiotics improved depressive-like behavior in CUMS mice, as indicated by decreased immobility time (*p* < 0.05) in the forced swimming test and tail suspension test. probiotics intervention also increased the level of 5-hydroxytryptamine (5-HT) in the prefrontal cortex and decreased the adrenocorticotropic hormone (ACTH) level in serum. In addition, by comparing the PFC gene expression among different groups, we found that the genes upregulated by probiotics were enriched in the PI3K-Akt signaling pathway in the prefrontal cortex. Moreover, we found that downregulated genes in prefrontal cortex of CUMS group such as *Sfrp5* and *Angpt2*, which were correlated with depression, were reversed by the probiotics. Furthermore, the probiotics altered the structure of the gut microbiota, and reversed the reduction of cob(II)yrinate a,c-diamide biosynthesis I pathway in CUMS group. Several species like Bacteroides caecimuris and Parabacteroides distasoni, whose abundance was significantly decreased in the CUMS group but reversed after the probiotics intervention, showed significantly positive correlation with depression associated genes such as *Tbxas1* and *Cldn2*.

**Discussion:** These findings suggested that CUMS-induced depression-like behavior can be alleviated by the probiotics, possibly through alterations in the PFC gene expression and gut microbiota.

## 1 Introduction

Major depressive disorder (MDD) is the most common mental disease in the world. According to the 2015 World Health Organization (WHO), the number of people living with MDD is expected to exceed 300 million, and MDD is also a major contributor to suicide deaths (Pitsillou et al., 2020). With MDD patients having primary disability and secondary disability from chronic medical illness, MDD is one of the most costly medical burdens in the world (Dean and Keshavan, 2017). Because of the effect of COVID-19, the number of patients worldwide has increased (COVID-19 Mental Disorders Collaborators, 2021). Stress is considered an internal threat to physiological homeostasis and psychological wellbeing. Moreover, stress is correlated with elevated morbidity and mortality, encompassing higher incidence rates of mental illness and suicide (Davis et al., 2017). Schmidt M V et al. and Belleau E L et al. provided hard evidence linking the experience of chronic stress to MDD (Schmidt et al., 2008; Belleau et al., 2019).

Despite the efforts of MDD research in the past few decades, the pathogenic mechanism has not been verified clearly, and the traditional drugs used for long-term treatment may induce side effects in patients, such as anorexia (Oliva et al., 2021), headache, sexual dysfunction (Giatti et al., 2018), bleeding, and even intracerebral hemorrhage.

Accumulating studies have shown an association between depression and dysbiosis of the gut microbiota. Compared with the healthy individuals, the gut microbial composition of MDD patients has changed, especially in terms of the diversity of the microbiota and the relative abundance of specific bacterial taxa (Jiang et al., 2015). Preclinical studies have verified that transplantation of the fecal microbiome from MDD patients into antibiotic-treated mice or rats induces anxiety and despair-like behavior (Zheng et al., 2016; Chevalier et al., 2020; Knudsen et al., 2021). Several probiotics have been found to be effective in the prevention or treatment of depression (Xu J. et al., 2022; Xu et al., 2022 M.). Therefore, probiotics, which are live microorganisms that, when administered in adequate amounts, confer health benefits to the host (Hill et al., 2014), are promising therapeutics to tackle MDD.

A complex probiotic of *Bacillus coagulans* CGMCC10182 and *Clostridium butyricum* CGMCC1647 was found to improve the mood of elderly individuals in the preliminary experiment. *Bacillus coagulans* has been used to treat gastrointestinal diseases, dental caries, and vaginitis (Mu and Cong, 2019). *Clostridium butyricum* is used to treat gastrointestinal diseases, metabolic diseases, cancer, and nerve disease (Stoeva et al., 2021). In the present study, we used a chronic unpredictable mild stress (CUMS) model to induce mice to develop depressive-like behaviors and explored the effects of the probiotics on neurotransmitters, hormones, depression-like behaviors, transcriptomics, and the gut microbiota.

## 2 Methods and materials

### 2.1 Animals and drugs

Six-week-old female BALB/c mice were housed once per cage at a temperature of 24°C ± 2°C, 40%–60% humidity, and a 12-h light/dark cycle. All animal experimental procedures were carried out according to the governmental guidelines and approved by the Ethics Committee on Laboratory Animals of the Shanghai Institute for Biomedical and Pharmaceutical Technologies (protocol code 2022-43, approval date 11/30/2022).

The probiotics used for intervention were a combination of *Bacillus coagulans* CGMCC10182 (5 × 10^8^ CFU/g) and *C. butyricum* CGMCC1647 (5 × 10^7^ CFU/g).

### 2.2 CUMS model

**FIGURE 1:**
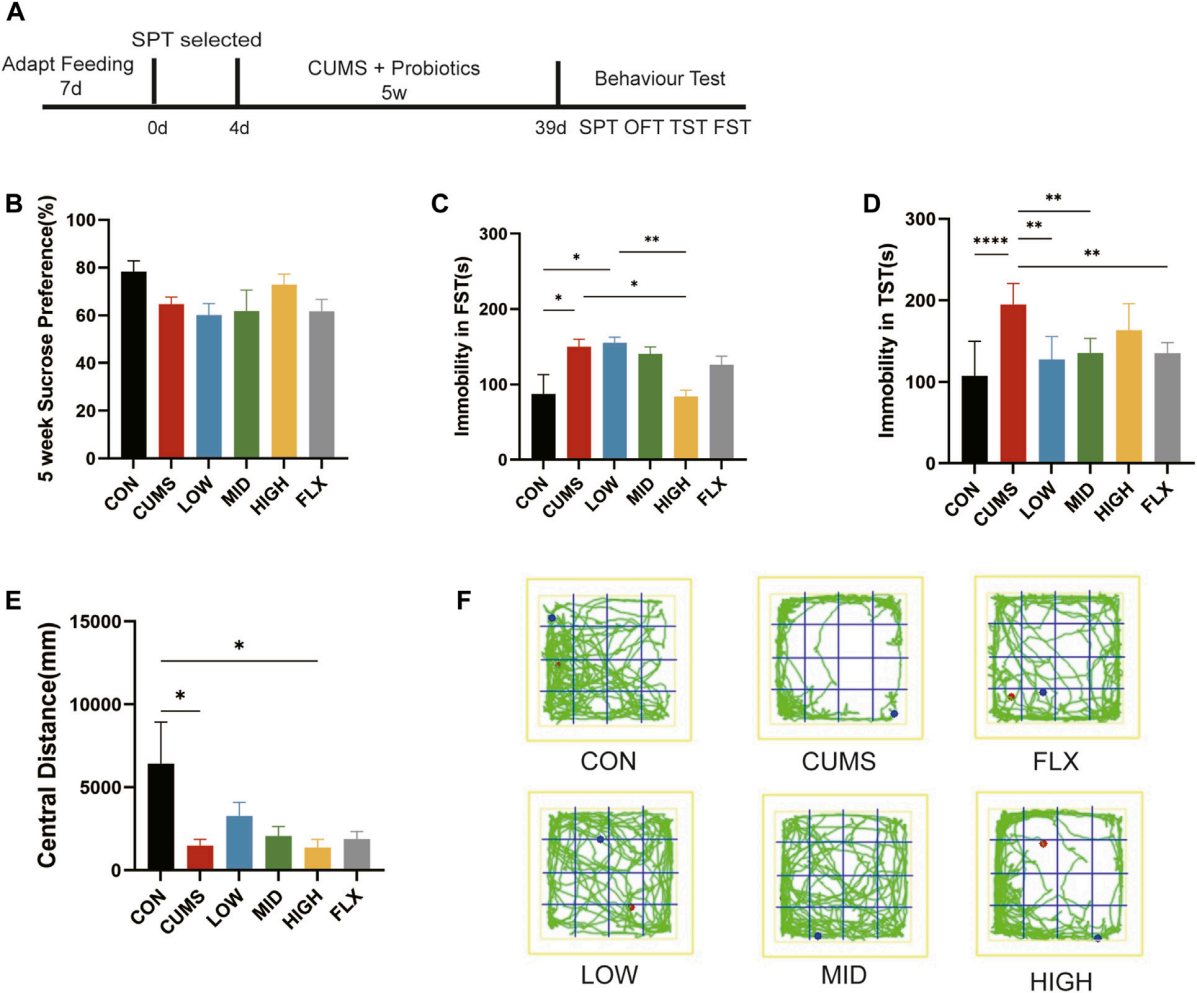
CUMS-induced depression-like behaviors were ameliorated by probiotics. **(A)** The treatment for mice. **(B)** SPT after 5 weeks treatment. **(C)** The forced swimming test (FST). **(D)** The Tail suspension test (TST). **(E)** The central distance of open field test (OFT). **(F)** The trails of mice in OFT. n = 7 in each group (*p < 0.05; **p < 0.01; ****p < 0.0001).

After one week of adjustable feeding, all mice were subjected to a 4-day sucrose preference test, and the mice whose sucrose preference was greater than 60% were selected for further experiments (Figure 1A). Forty-two selected BALB/c mice were divided into six groups: the control group (CON), model group (CUMS), low-concentration complex probiotics group (LOW), middle-concentration complex probiotics group (MID), high-concentration complex probiotics group (HIGH), and fluoxetine group (FLX). In addition to the CON group, the other groups were subjected to CUMS for 5 weeks by exposing them to two out of eight treatments each day: clamping of the tail (3 min), swimming in 4°C water (5 min), restraining in 50 mL tubes (3 h), crowded feeding (24 h), water deprivation (18 h), food deprivation (20 h), empty cage (24 h), tilted cage (24 h), damp cage (24 h), and darkness (36 h). The two stimuli were randomly selected per day and not applied the next day, but all the mice in the five groups were subject to the same stimuli per day to avoid difference.

During CUMS treatment, the three probiotic treatment groups received complex probiotics at 13 mg/kg (LOW), 52 mg/kg (MID), and 204 mg/kg (HIGH) once a day, which was determined by referring to the usage dose of *B*. *coagulans* in clinical trials (Zhang et al., 2023) and *C*. *butyricum* across murine models (Stoeva et al., 2021). The model group (CUMS) received the same volume of PBS, and the FLX group received fluoxetine 30 mg/kg once a day (Taguchi et al., 2016).

### 2.3 Sucrose preference test

The sucrose preference test (SPT) can be used to assess depression in mice. The degree to which the mice preferred sugar water was inversely proportional to the degree of depression. This test was designed based on the SPT protocol (Liu M.-Y. et al., 2018). All mice were housed in a single cage, acclimated for 24 h, and then fasted and water deprived for 24 h. Each mouse was given one bottle of 1% sucrose solution and one bottle of sterile water. The consumption of sucrose solution and sterile water was recorded for 24 h (changing the position after 12 h), after which the sucrose preference rate was calculated. The sugar preference experiment was performed at weeks 0 and 5. Initially, mice with a sucrose preference greater than 60% were selected for modeling, as a sucrose preference <60% is defined as no preference in normal mice (Liu M.-Y. et al., 2018). In the fifth week, only the sugar-water test was performed on all the model mice.

### 2.4 Behavior test

#### 2.4.1 Open field test

This experiment was designed by a previous study (Kraeuter et al., 2019). The open field served as a platform measuring 500*500*400 mm, allowing mice unrestricted movement for observing their behavior. A correlation was observed between the mice’s level of anxiety and the reduced time spent in the central region. All mice were consistently placed in the center of the open field arena, facing the same direction. Subsequently, behavior video recording and analysis software was used to record the movement of the animals within the open field for 5 min.

#### 2.4.2 Tail suspension test

The tail suspension test (TST) was used to test the depressive-like behavior in mice. The TST was designed based on the previous method (Castagné et al., 2011). Medical tape was used to place the mice’s tails 50 cm above the floor at the same location. After each test, a 75% ethanol solution was used to wipe the field. The immobility time was recorded after 4 min for 6 min.

#### 2.4.3 Forced swimming test

Forced swimming tests (FST) were also used to test depressive-like behavior in mice. This test was referred to the previous study (Yankelevitch-Yahav et al., 2015). Each mouse was put in a 40 cm high cylinder with a 15 cm diameter. The apparatus was filled with 24°C water to a height of 15 cm. The mice were forced to swim in the cylinder for 6 min, and immobility in the last 4 min was calculated.

### 2.5 Serum and tissue collection

After 5 weeks of treatment, the mice were sacrificed, and blood samples collected from the heart were centrifuged at 3,000 rpm for 10 min. The supernatant was transferred to a new microcentrifuge to obtain the serum, which was stored at −80°C. Half of brain was fixed with 4% (w/v) paraformaldehyde for histopathological staining. And the prefrontal cortex (PFC) from the other part of the brain was isolated and stored at −80°C after flash freezing in liquid nitrogen for biochemical tests and transcriptome sequencing. Feces were collected in sterile tubes and then stored at −80°C.

### 2.6 Enzyme-linked immunosorbent assay (ELISA)

The levels of inflammatory neurotransmitters and hormones in serum and PFC were measured by ELISA. The PFC were rinsed with precooled PBS (0.01 M, pH = 7.4) and cut into pieces after weighing. The trimmed PFC was homogenized in PBS (1:9 weight-to-volume ratio, and a protein inhibitor was added to PBS), the homogenate was centrifuged at 5,000 × g for 10 min, and the supernatant was stored at −80°C. Briefly, 100 μL of standard or sample was added to each well and incubated for 90 min at 37°C. Then, 100 μL of biotinylated detection antibody working solution was added to each well, and the plates were incubated for 60 min at 37°C. After the plates were aspirated, they were washed 3 times. A total of 100 μL of HRP conjugate working solution was added. The mixture was incubated for 30 min at 37°C. The plate was aspirated and washed 5 times. Subsequently, 90 μL of TMB substrate reagent was added, and the mixture was incubated for 15 min at 37°C. After the incubation, 50 μL of stop solution was added to each well to stop the reaction. Finally, the absorbance at 450 nm was measured immediately and calculated. The levels of adrenocorticotropic hormone (ACTH), corticosterone (CORT), and 5-hydroxytryptamine (5-HT) were determined using ELISA kits (Elabscience, China), and the level of brain-derived neurotrophic factor (BDNF) was determined using an ELISA kit (Boster, China).

### 2.7 Nissl staining

The half brain was immersed in 4% paraformaldehyde solution for 24–48 h, stored, and transported at room temperature. The brain was removed from the fixative, and a scalpel was used to smooth the target tissue in the fume hood. The cut tissue and the corresponding label were placed in the embedding frame. Then, the dehydration box was placed in a dehydrator for dehydration with gradient alcohol. The following steps were used: 75% alcohol for 4 h, 85% alcohol for 2 h, 90% alcohol for hours, 95% alcohol for 1 h, anhydrous ethanol for 30 min, anhydrous ethanol for 30 min, alcohol for benzene for 5–10 min, xylene I for 5–10 min, 65°C melting paraffin I for 1 h, 65°C melting paraffin II for 1 h, and 65°C melting paraffin for 1 h. Next, the wax-soaked tissue was embedded in the embedding machine. First, the melted wax was placed into the embedding frame, and before the wax solidified, the tissue was removed from the dewatering box and put into the embedding frame according to the requirements of the embedding surface, and the corresponding label was affixed. After the wax solidified, the wax block was removed from the embedding frame and repaired. The trimmed wax blocks were placed into a paraffin slicer for slicing at a thickness of 4 μM. The tissue was flattened when the slice floated on the 40°C warm water of the spreading machine, and the tissue was picked up by glass slides and baked in an oven at 60°C. After the water-baked dried wax was melted, it was removed and stored at room temperature.

The paraffin sections were sequentially immersed in environmentally friendly dewaxing transparent liquid I for 20 min, environmentally friendly dewaxing transparent liquid II for 20 min, anhydrous ethanol I for 5 min, anhydrous ethanol II for 5 min, and 75% ethyl alcohol for 5 min and then rinsed with tap water. The tissue slices were treated with dye solution for 2–5 min and rinsed with tap water. Then, 0.1% glacial acetic acid was used for slight differentiation, the reaction was terminated by washing with running water, and the degree of differentiation was controlled under a microscope. The sample was washed with tap water and dried in an oven.

### 2.8 Transcriptome sequencing

#### 2.8.1 RNA extraction

Total RNA was extracted from three PFC tissues of each group, except that MID collected two sample, as a total of 17 samples using TRIzol^®^ Reagent according to the manufacturer’s instructions. Then, RNA quality was determined by 5,300 Bioanalyser (Agilent) and quantified using the ND-2000(NanoDrop Technologies). Only high-quality RNA samples (OD260/280 = 1.8–2.2, OD260/230 ≥ 2.0, RIN≥6.5, 28S:18S ≥ 1.0, >1 μg) were used to construct the sequencing library.

#### 2.8.2 Library preparation and sequencing

RNA purification, reverse transcription, library construction, and sequencing were performed at Shanghai Majorbio Biopharm Biotechnology Co., Ltd. (Shanghai, China) according to the manufacturer’s instructions (Illumina, San Diego, CA). The RNA-seq transcriptome library was prepared following Illumina^®^ Stranded mRNA Prep, Ligation from Illumina (San Diego, CA) using 1 μg of total RNA. Briefly, messenger RNA was first isolated by oligo(dT) beads according to the poly(A) selection method and then fragmented by fragmentation buffer. Second, double-stranded cDNA was synthesized using a SuperScript double-stranded cDNA synthesis kit (Invitrogen, CA) with random hexamer primers (Illumina). Then, the synthesized cDNA was subjected to end-repair phosphorylation and ‘A’ base addition according to Illumina’s library construction protocol. Libraries were size selected for cDNA target fragments of 300 bp on 2% low-range Ultra agarose followed by PCR amplification using Phusion DNA polymerase (NEB) for 15 PCR cycles. After quantification by Qubit 4.0, the paired-end RNA-seq library was sequenced with a NovaSeq Xplus sequencer (2 × 150 bp read length).

#### 2.8.3 Quality control and read mapping

The raw paired-end reads were trimmed and quality controlled by fastp (Chen et al., 2018) with default parameters. Then, the clean reads were separately aligned to the reference genome in orientation mode using HISAT2l (Kim et al., 2015) software. The mapped reads of each sample were assembled by StringTie (Pertea et al., 2015) via a reference-based approach.

#### 2.8.4 Differential expression analysis and functional enrichment

To identify differentially expressed genes (DEGs) between different samples, the expression level of each transcript was calculated according to the transcripts per million reads (TPM) method. RSEM (Li and Dewey, 2011) was used to quantify gene abundances. Essentially, differential expression analysis was performed using DESeq2 (Love et al., 2014). DEGs with |log2FC| ≥ 0.585 and *p*-value < 0.05 (DESeq2) were considered to be significantly differentially expressed genes. In addition, functional enrichment analysis, including GO and KEGG enrichment analyses, was performed to identify which DEGs were significantly enriched in GO terms and metabolic pathways at a Bonferroni-corrected *p*-value < 0.05 compared with the whole-transcriptome background. GO functional enrichment and KEGG pathway analyses were carried out by Goatools and Pythonscipy, respectively. All the data were analyzed on the Majorbio Cloud Platform (https://cloud.majorbio.com/) (Ren et al., 2022).

### 2.9 16S rRNA gene sequencing

Fecal samples from each mouse were collected at the end of the 5-week treatment before sacrifice, and 42 samples were collected in total. A QIAamp DNA Stool Mini Kit (QIAGEN) was used for bacterial genomic DNA extraction. For the detection of 16S rRNA genes, the V3-V4 region was amplified with the primers 338F and 806R (Huse et al., 2007) using TransStart Fastpfu DNA Polymerase (TransGen). The thermocycling steps were as follows: 95°C for 5 min, 20 cycles at 95°C for 45 s, 55°C for 30 s, 72°C for 30 s, and a final extension step at 72°C for 10 min. Three repeat PCR amplifications of each sample were performed, and then all amplicons were purified with an AxyPrep DNA Gel Extraction Kit (AXYGEN) and pooled equivalent after being assessed by spectrophotometry (QuantiFluor-ST, Promega). Sequencing of 16S rRNA gene amplicons was performed on an Illumina NextSeq 2000 instrument (2 × 300 bp).

QIIME 2 (version 2023.9) (Bolyen et al., 2018) was used to analyze the microbiota data. DADA2 of the QIIME 2 plugin was used for quality control and ASV exaction with default parameters. The minimum sample size was the criteria for data normalization. Community richness, evenness, and diversity analysis (Shannon, Simpsonenven, ACE, Chao, and Good’s coverage) were performed using Mothur (Schloss et al., 2009). Taxonomy was assigned using the software RDP classifier (Wang et al., 2007) at the default parameter (80% threshold) based on the Ribosomal Database Project (Cole et al., 2009). Species assignment was performed by BLASTN against the SILVA reference database (version 138.1) (Quast et al., 2012) with the following parameters: the highest score, identity >97%, and alignment >97%. Differences between groups were assessed using ANOSIM and PerMANOVA in PAST (version 4.16c). Principal coordinate analysis (PCoA) was based on the Bray‒Curtis distance matrix. LEfSe (Segata et al., 2011) used the Kruskal‒Wallis test to detect differentially abundant taxa (*p* < 0.05) between the two groups and estimate the linear discriminant analysis effect size (LDA score >2.0). By normalizing the 16S rRNA gene copy numbers, PICRUSt2 (Douglas et al., 2020) was used to predict the microbiome functions based on the MetaCyc pathways. The difference in predicted functions was analyzed using STAMP (Parks et al., 2014) with default parameters.

The correlation analysis between the transcriptome and gut microbiota was performed through a nonparametric Spearman rank correlation algorithm, by calculating the correlation coefficient between the abundance of species and TPM of genes, and the parameters were set as a coefficient >0.68 or −0.68 and FDR<0.05, which were considered to represent high correlations.

### 2.10 Statistical analysis

Statistical analyses were conducted by using GraphPad Prism 9. All values are expressed as the means ± standard errors of the means. All the data were tested using one-way ANOVA. *p* < 0.05 was considered to indicate statistical significance.

## 3 Results

### 3.1 CUMS-induced depression-like behaviors were ameliorated by probiotics

To investigate the effect of treatment on CUMS-induced depression-like behaviors, the SPT, TST, FST, and OFT were used to detect depression and antidepression-like behaviors in mice.

After five weeks of treatment, sucrose preference did not show a significant difference among the six groups in SPT (Figure 1B), though the CUMS group showed a decreasing trend relative to CON and the HIGH group showed an increasing trend, which indicated that neither CUMS stimulation nor the probiotics or fluoxetine influenced anhedonia.

CUMS stimulation significantly increased immobility time in the FST compared with that in the CON group (Figure 1C, *p* < 0.05), and the high concentration of probiotics reduced immobility time to the CON level, showing a greater amelioration effect than did Fluoxetine (Figure 1C).

Moreover, as the TST showed, the immobility time of the CUMS group in TST was significantly greater than that of the CON group (Figure 1D, *p* < 0.0001). Treatment with low- and mid-concentration probiotics and Fluoxetine notably reduced immobility time (*p* < 0.01). The TST and FST showed that CUMS can lead to depression-like behavior in mice, and our treatment with different concentrations of the probiotics improved CUMS-induced despair-like behavior.

The OFT was used to evaluate the anxiety-like behavior of the mice, and the CUMS-treated mice exhibited significantly reduced activity in the central region compared with that in the CON group (Figures 1E, F, *p* < 0.05), suggesting that the CUMS-exposed mice exhibited anxiety-related behaviors. Although not significant, the central distance tended to improve in the LOW and MID groups compared with that in the CUMS group. These results suggested that low and middle concentrations of the probiotics may improve CUMS-induced anxiety.

### 3.2 Effects of probiotics on neurotransmitter and hormone levels and morphological changes in the hippocampus and prefrontal cortex

**FIGURE 2:**
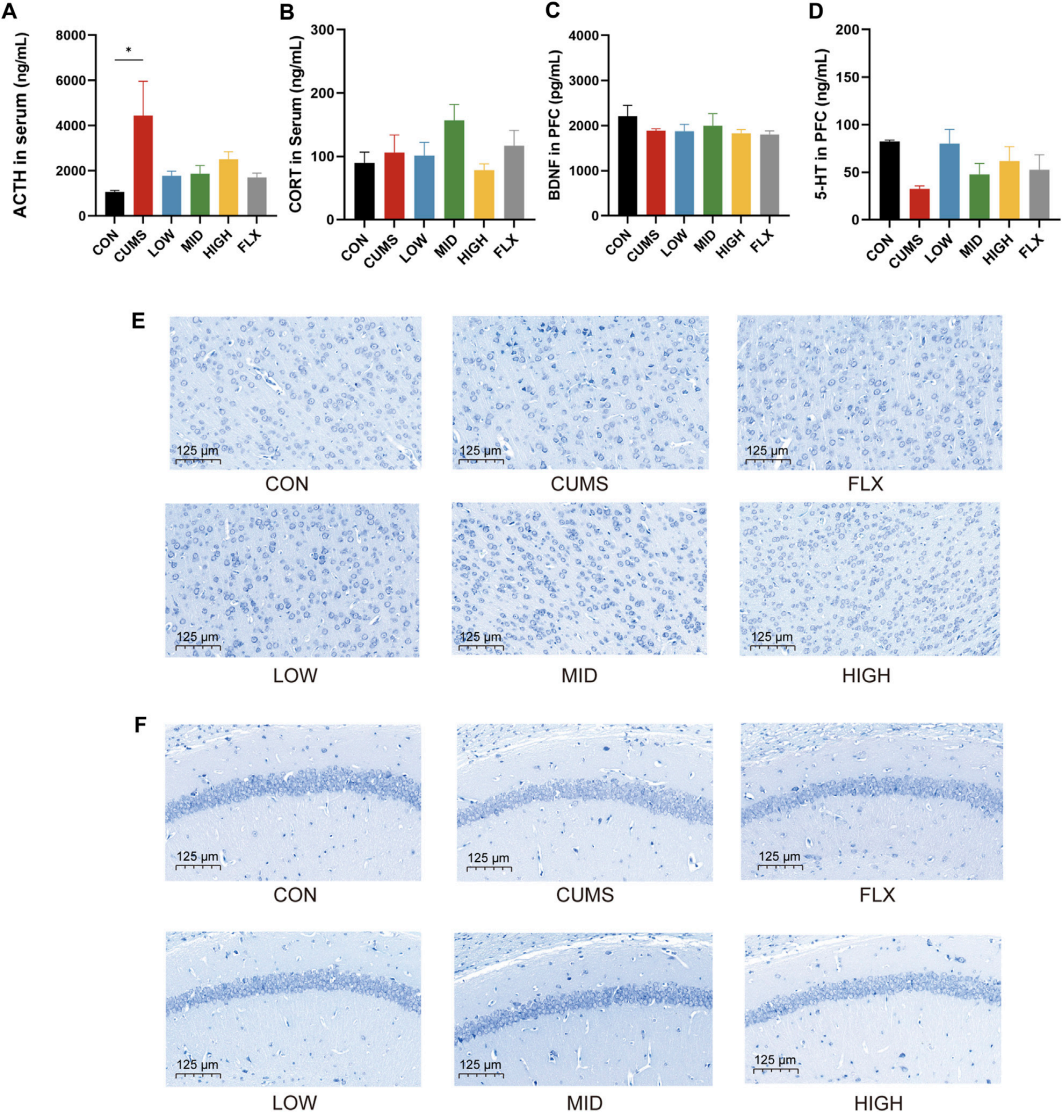
Effects of probiotics on neurotransmitter and hormone and morphological changes of hippocampi and cortex. **(A)** The level of ACTH in serum (*n* = 3, *p < 0.05). **(B)** The level of CORT in serum, *n* = 3. **(C)** BDNF level in the prefrontal cortex (PFC), *n* = 3. **(D)** 5-HT level in PFC, *n* = 3. **(E)** Nissl staining of neurons in PFC. **(F)** Nissl staining of hippocampus. Scale bar = 125 μm.

CUMS significantly increased the serum ACTH concentration (Figure 2A, *p* < 0.05), and the probiotics tended to reduce the level of ACTH in serum. However, the levels of CORT in the serum (Figure 2B) and BDNF (Figure 2C) in the prefrontal cortex (PFC) showed no significant differences among the CON group, CUMS group, and probiotics treatment groups. Moreover, there was a noticeable decreasing trend in the 5-HT concentration in the PFC following CUMS, and various concentrations of the probiotics increased the 5-HT concentration to varying degrees compared to that in the CUMS group (Figure 2D).

Nissl staining was applied to track the morphological alterations of neurons in the cortex and hippocampus. As shown in Figure 2E, the cortex region of the CON group showed normal, numerous, and compactly arranged neurons. In contrast, the CUMS group exhibited a diminished quantity of neurons, along with irregular neuronal arrangement, nuclear shrinkage, and indistinct nuclear boundaries. The probiotic treatment reversed the effects of CUMS treatment. All concentrations of probiotics significantly reduced these signs of histopathological damage.

In addition, the neurons in the hippocampal CA1 region were sparsely distributed and exhibited a reduced population compared to those in the CON group (Figure 2F), while the number of Nissl bodies was notably increased in the CON group and the probiotics group.

### 3.3 Transcriptomic profiles of mouse PFC under CUMS and probiotics treatment

**FIGURE 3:**
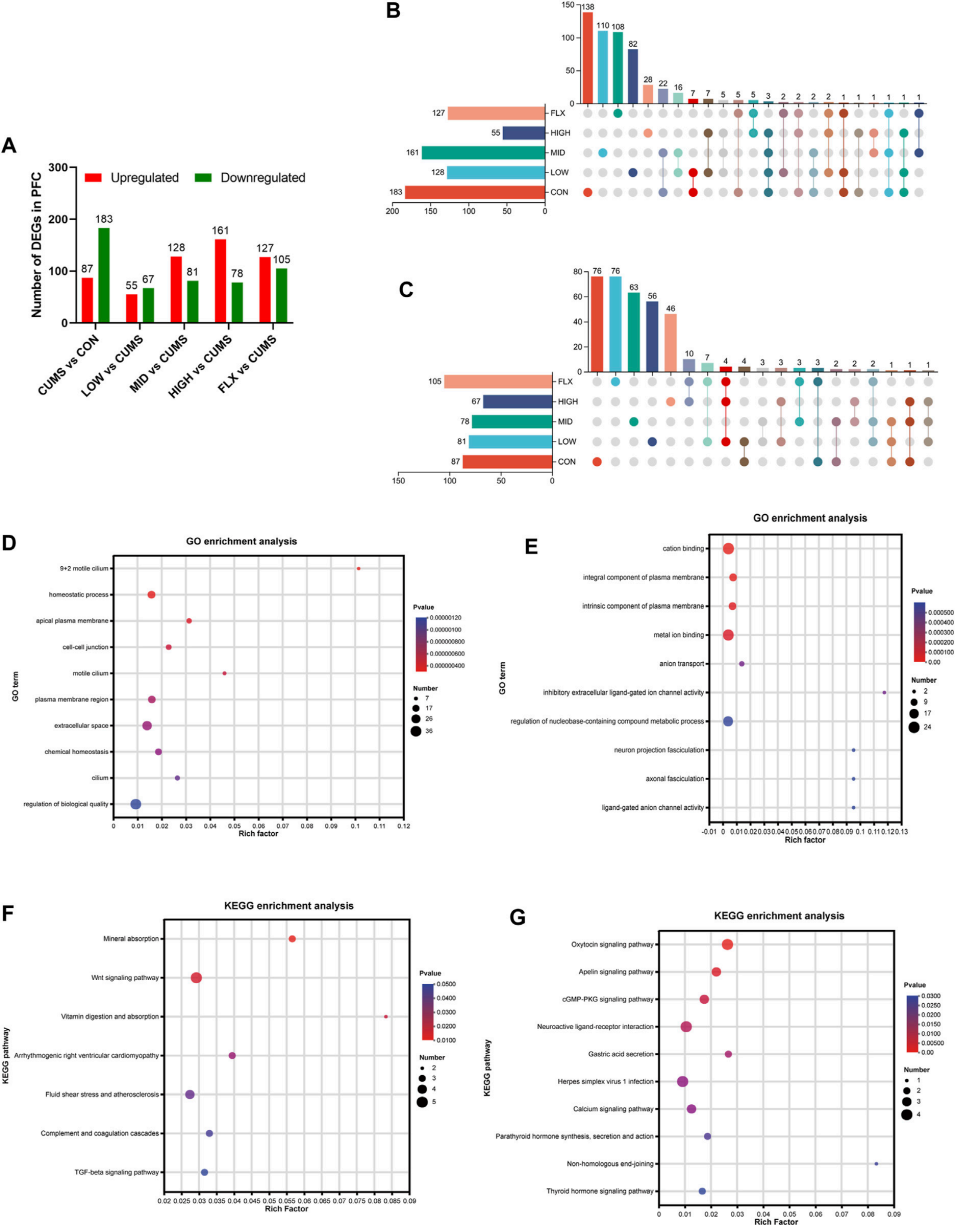
Transcriptome data of mice among the different groups. **(A)** Analysis of DEGs number in the PFC. **(B)** Upset plot for downregulated genes in the CUMS group which were reversed after probiotics or FLX intervention. **(C)** Upset plot for upregulated genes in the CUMS group which were reversed after Probiotics or FLX intervention. **(D)** Go enrichment analysis of downregulated genes in CUMS group. **(E)** Go enrichment analysis of downregulated genes in CUMS group. **(F)** KEGG enrichment analysis of downregulated genes in the CUMS group. **(G)** KEGG enrichment analysis of upregulated genes in the CUMS group.

To investigate the potential molecular mechanisms underlying the ameliorative effect of the probiotics on the CUMS model, transcriptomic analysis was carried out on the PFC of mice from the six groups. Compared with those in the CON group, 270 DEGs were identified in the CUMS group, with 87 upregulated genes and 183 downregulated genes (Figure 3A). The top 10 GO terms enriched for the downregulated genes in the CUMS group are shown in Figure 3D, most of which were associated with cellular components. The top 10 GO terms enriched for the upregulated genes in the CUMS group are shown in Figure 3E, such as anion transport (GO:0006820), neuron projection fasciculation (GO:0106030), and axonal fasciculation (GO:0007413). KEGG pathway enrichment analysis confirmed the impact of CUMS treatment on specific biological pathways. The downregulated genes after CUMS treatment were enriched in several pathways such as the Wnt signaling pathway and the TGF-beta signaling pathway (Figure 3F), while the upregulated genes were enriched in the cGMP-PKG signaling pathway, Neuroactive ligand-receptor interaction pathway, and Calcium signaling pathway, etc. (Figure 3G).

In addition, enrichment analysis of DEGs in each intervention group (LOW, MID, HIGH, and FLX) relative to CUMS was performed. The upregulated genes in both the LOW and HIGH groups were significantly enriched in the PI3K-Akt signaling pathway (Supplementary Figure S1).

Additionally, a comprehensive analysis was conducted to delineate the DEGs of CUMS group that were reversed by different concentrations of probiotics (Figures 3B, C; Tables 1, 2). We identified several genes related to the nervous system or depression, such as *Sfrp5* and *Angpt2*. *Sfrp5* was downregulated in the CUMS group and reversed by mid concentration of probiotics significantly (Table 1; Supplementary Table S1). Moreover, *Angpt2* was downregulated in the CUMS group and reversed by high concentration of the probiotics and fluoxetine significantly.

**TABLE 1:** The CUMS downregulated genes reversed by probiotics.

Group	Gene name
LOW	*Mirt1, Pomc, Nkd2, Slc9a4, Pkp2, Tspan18, Serpina3g*
MID	*Fmod, Sfrp5, Hif3a, Gm46223, Mfrp, Trpv4, F5, Steap1, Krt8, Gm45090, Slc2a12, Angptl2, Krt18, Tnfaip6, Cfap65, Cfap95, Stard13, Ltc4s, Tuba1c, Slc13a4, Dnai3, Cox8b*
HIGH	*Tekt4*
LOW and MID	*Scnn1a, Maff*
LOW and HIGH	*Fam163a*
LOW, MID and HIGH	*Plin4, Gm 2042, Rpl35a-ps4*
LOW and FLX	*Thbs4*
MID and FLX	*Steap4*
HIGH and FLX	*Gstp2, Angpt2*

Italic means the gene name of mice

**TABLE 2:** The CUMS upregulated genes reversed by probiotics.

Group	Gene name
LOW	*Gm7233, Olfr464, Gm11578, Gm6397*
MID	*Fam220a, Ccdc160*
LOW and MID	*Gm3604*
MID and HIGH	*Pagr1b*
LOW, MID and HIGH	*Gm17122*
LOW and FLX	*Gm8325, Mup11, Rpl14-ps1, Gm36981, Gm44873, Zglp1, Gm2446*
MID and FLX	*Rps12-ps3, Rpl35a-ps3, Gm8226*
HIGH and FLX	*Nr4a3, Egr2, Egr1, Egr4, Arc, Junb, Gm10221, Ccn1, Gm4988, Gm49936, Tnfrsf25*
LOW, HIGH and FLX	*Gm26736, Lrrc71, Col2a1, Gm10434*
LOW, MID and FLX	*Mup14, Cd300ld5*

Italic means the gene name of mice

For the DEGs in the CUMS group whose expression were reversed by probiotics treatment, the enrichment analysis revealed that they were mainly enriched in the GO terms such as sodium ion import across plasma membrane (GO:0098719), glutathione peroxidase activity (GO: GO:0004602), glutathione transferase activity (GO:0004364), etc. (Table 3).

**TABLE 3:** The GO enrichment analysis of DEGs in the CUMS group whose expression were reversed by probiotics.

GO term	Description	Genes
GO:0071944	cell periphery	*Krt18, Tspan18, Krt8, Nkd2*
GO:0005615	extracellular space	*Pomc, Angpt2, Krt18, Tnfaip6, Sfrp5, Angptl2, Fmod, Serpina3g, Thbs4, F5*
GO:0006811	ion transport	*Slc9a4, Steap4, Trpv4, Scnn1a, Steap1*
GO:0005882	intermediate filament	*Krt18, Krt8, Pkp2*
GO:0006814	sodium ion transport	*Slc9a4, Slc13a4, Scnn1a*
GO:0050891	multicellular organismal water homeostasis	*Trpv4, Scnn1a*
GO:0043508	negative regulation of JUN kinase activity	*Gstp2, Sfrp5*
GO:0005576	extracellular region	*Pomc, Angpt2, Tnfaip6, Sfrp5, Angptl2, Fmod, Thbs4, F5*
GO:0005929	cilium	*Trpv4, Scnn1a, Tekt4, Cfap65*
GO:0097284	hepatocyte apoptotic process	*Krt18, Krt8*
GO:0034755	iron ion transmembrane transport	*Steap4, Steap1*
GO:0098719	sodium ion import across plasma membrane	*Slc9a4, Scnn1a*
GO:0005886	plasma membrane	*Steap4, Angpt2, Cfap95, Gstp2, Slc2a12, Hif3a, Nkd2, Slc9a4, Mfrp, Krt18, Trpv4, Scnn1a, Pkp2, Plin4, Steap1, Cfap65*
GO:0031514	motile cilium	*Scnn1a, Tekt4, Cfap65*
GO:0016324	apical plasma membrane	*Slc9a4, Mfrp, Trpv4, Scnn1a*
GO:0060294	cilium movement involved in cell motility	*Dnai3, Tekt4*
GO:0004602	glutathione peroxidase activity	*Gstp2, ltc4s*
GO:0042995	cell projection	*Angpt2, Cfap95, Trpv4, Scnn1a, Tekt4, Cfap65*
GO:0004364	glutathione transferase activity	*Gstp2, Ltc4s*

Italic means the species of bacteria

### 3.4 Gut microbiota analysis

#### 3.4.1 Changes in the gut microbiota in the CUMS and intervention groups

To investigate the gut microbiota changes after CUMS treatment and probiotics or FLX intervention, we collected 42 fecal samples from the six groups. A total of 1,380,576 (26,043–39,198) high-quality 16S rRNA genes were obtained by high-throughput DNA sequencing. To avoid statistical bias, 26,043 was chosen as a normalization size for each sample. A total of 2,867 ASVs (684–1,323 in each group) were obtained. The Good’s coverage was greater than 99.99% for each sample, which meant that the sequencing depth was sufficient for gut microbiota investigation.

**FIGURE 4:**
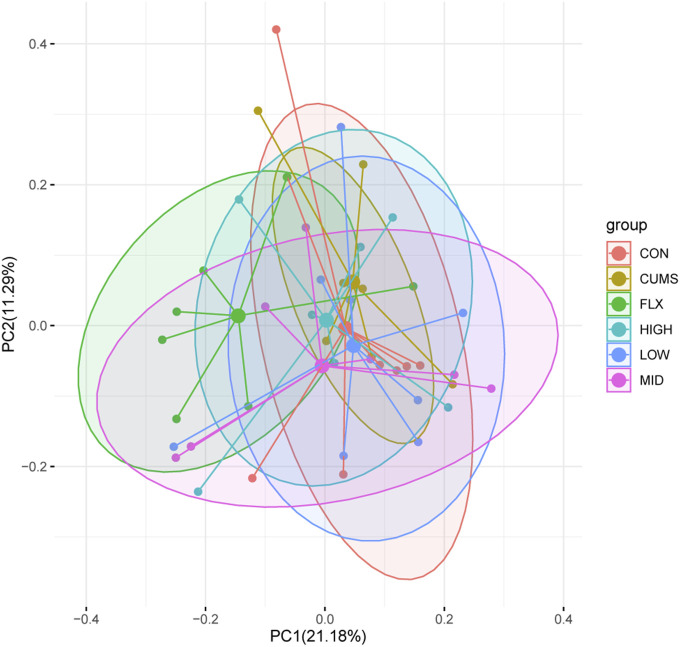
Principal coordinate analysis (PCoA) of gut microbiota among the six groups based on Bray Curtis distance matrix.

After taxonomic assignment, all 16S rRNA genes could be aligned to nine phyla, 98 genera, and 131 species. Through ANOSIM and PerMANOVA analysis, a significant difference was found in gut microbiota structure between CUMS group and other groups (Figure 4; Supplementary Table S2; Supplementary Table S3). The results revealed that the microbiota structures of the CUMS group were significantly different from those of the CON group, and the FLX and probiotics interventions significantly altered the microbiota structures of the CUMS group, while the MID group showed no significant difference from the CON group. There was no significant difference among the probiotics groups (including the LOW, MID, and HIGH groups), but the middle doses showed the best effect on restoring the gut microbiota composition to normal status (*p* = 0.054), followed by the high dose (*p* = 0.393).

#### 3.4.2 Gut microbiota characteristics of the CUMS group

**FIGURE 5:**
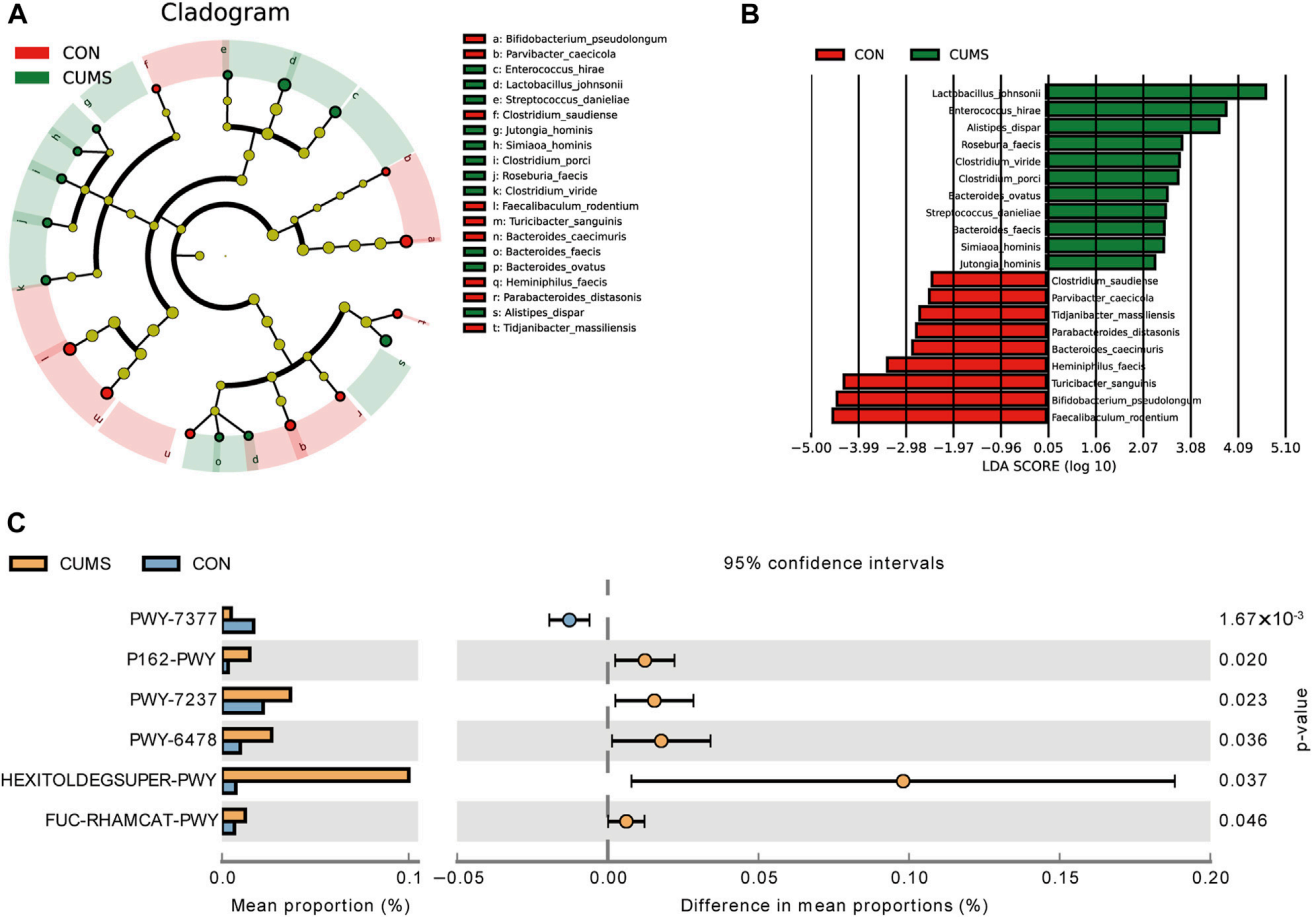
Comparison of the bacterial taxa in the gut microbiota of CUMS and CON groups. **(A)** A cladogram taxonomic representation representing distinct bacterial taxa between the two groups. **(B)** A histogram of the linear discriminant analysis (LDA) scores represents significant differences in the abundance of the bacterial taxa between the two groups. **(C)** Difference in functional pathway prediction using PICRUSt for gut microbiota.

LEfSe analysis revealed that 20 species exhibited significant changes (*p* <0.05) in the CUMS group compared with the CON group (Figures 5A, B), including nine decreased species and 11 increased species in the CUMS group, such as *Bifidobacterium pseudolongum* (0.48% in CUMS, and 5.99% in CON) and *Alistipes dispar* (2.11% in CUMS, and 1.02% in CON). Meanwhile, the relative abundance of 31 species was significantly changed in the probiotics groups relative to CUMS group (Supplementary Figure S2), and 14 species were significantly changed in the FLX group relative to the CUSM group (Supplementary Figure S3). Among the nine species whose abundance decreased in the CUMS group relative to that in the CON group, five were significantly increased (*p* < 0.05) after probiotics intervention and three were significantly increased after FLX intervention (Table 4). Although showing no significance, the probiotics showed a trend in increasing the abundance of *B*. *pseudolongum* (0.76%–1.7%). Among the 11 increased species in the CUMS group, nine were significantly decreased (*p* < 0.05) after probiotics intervention and two were significantly decreased after FLX intervention (Table 4).

**TABLE 4:** Significantly changed species in CUMS group and the abundance changes after intervention.

Species	Enriched in	CON (%)	CUMS (%)	LOW	MID	HIGH	FLX
*Bacteroides caecimuris*	CON	0.14	0.0062	0.095%	0.16%*	0.20%*	0.23%*
*Bifidobacterium pseudolongum*	CON	5.99	0.48	1.220%	1.70%	0.76%	0.15%
*Clostridium saudiense*	CON	0.032	0.00	0.0034%	0.00%	0.00%	0.0016%
*Faecalibaculum rodentium*	CON	7.64	0.00	0.14%	0.00%	0.00%	0.00%
*Heminiphilus faecis*	CON	0.55	0.0072	2.05%*	0.54%*	1.04%	0.18%
*Parabacteroides distasonis*	CON	0.12	0.00	0.18%*	0.091%*	0.11%*	0.00%
*Parvibacter caecicola*	CON	0.027	0.00	0.015%	0.00%	0.034%*	0.021%*
*Tidjanibacter massiliensis*	CON	0.10	0.0029	0.17%*	0.16%*	0.20%*	0.31%*
*Turicibacter sanguinis*	CON	4.270	0.036	1.15%	3.35%	0.43%	0.0082%
*Clostridium viride*	CUMS	0.014	0.14	0.038%#	0.054%#	0.048%#	0.078%
*Alistipes dispar*	CUMS	1.020	2.11	1.00%#	1.14%#	1.73%	2.41%
*Bacteroides faecis*	CUMS	0.00	0.05	0.00%#	0.00%#	0.00%#	0.00%#
*Bacteroides ovatus*	CUMS	0.00	0.064	0.00%#	0.00%#	0.00%#	0.00%#
*Clostridium porci*	CUMS	0.055	0.16	0.10%	0.051%	0.028%	0.36%
*Enterococcus hirae*	CUMS	0.0024	1.52	3.07%	6.39%	2.40%	0.80%
*Jutongia hominis*	CUMS	0.00	0.017	0.0019%#	0.00%#	0.0022%	0.024%
*Lactobacillus johnsonii*	CUMS	0.00	8.40	0.026%#	0.05%#	0.028%#	3.61%
*Roseburia faecis*	CUMS	0.0014	0.12	0.00%#	0.021%#	0.011%#	0.11%
*Simiaoa hominis*	CUMS	0.00	0.045	0.00%#	0.00%#	0.00%#	0.076%
*Streptococcus danieliae*	CUMS	0.012	0.068	0.024%#	0.069%	0.044%	0.12%

*Significantly increased (*p* <0.05) compared with CUMS.

# significantly decreased (*p* <0.05) compared with CUMS.

#### 3.4.3 Functional prediction of gut microbiota and the correlation between the transcriptome and gut microbiota

The potential functions of the microbiota were predicted, and six pathways exhibited significant changes between the CUMS group and the CON group (Figure 5C; Supplementary Table S4). The pathway PWY-7377, cob(II)yrinate a c-diamide biosynthesis I (early cobalt insertion), was significantly decreased in CUMS group. The other five pathways were all increased in CUMS group, such as the super pathway of fucose and rhamnose degradation (FUC-RHAMCAT-PWY), and myo-, chiro- and scylla-inositol degradation (PWY-7237).

A total of 45 pathways exhibited significant changes between the probiotics group and the CUMS group (Supplementary Table S4). The PWY-7377 pathway, which was decreased in the CUMS group, was significantly increased in probiotics groups (LOW and HIGH) compared with the CUMS group. Though 80 pathways were significantly changed after FLX intervention the PWY-7377 pathway showed no significant change in the FLX group.

We then performed a correlation analysis between the transcriptome and the gut microbiota. A total of 145 positive correlations and 92 negative correlations at the genus level (Supplementary Table S5), and there were 146 positive correlations and 77 negative correlations at the species level (Supplementary Table S6). We found that *Bacteroides caecimuris* was positively related to *Tbxas1* (ENSMUSG00000029925), and *Parabacteroides distasonis* was positively related to *Cldn2* (ENSMUSG0000004723). Both genes showed a decrease trend after CUMS and increased by probiotics intervention (Supplementary Table S7).

## 4 Discussion

Nowadays, MDD is receiving increasing amounts of attention worldwide. Our current research investigated the effects of probiotics, a combination of *Bacillus coagulans* and *C. butyricum*, by using CUMS-induced depression mouse model. In addition, we sought to identify the potential pathways underlying the effect of probiotics through transcriptome and gut microbiota analysis.

*Lactobacillus rhamnosus,* a well-known probiotic, has a significant effect on alleviating depression behavior in CUMS models and MDD patients (Rudzki et al., 2019; Xu J. et al., 2022; Xu M. et al., 2022). Most previous studies have investigated the effects of *Bacillus coagulans or C. butyricum* alone on depression (Zhang W. et al., 2022; Satti et al., 2023). Our study is the first to use the combination of *Bacillus coagulans* and *C. butyricum* to determine the effect of antidepressant-like capacity.

The CUMS mouse model is the most commonly used depression model and can mimic stressful experiences in humans (Liu S. et al., 2018; Antoniuk et al., 2019; Zhang Y. et al., 2022). The strain of mice with BALB/c and C57BL/6J were both used for depression. A previous study has shown that the BALB/c mice have higher sensitivity than C57BL/6J to environmental stressors under CUMS model (Malki et al., 2015).

There is no difference in anhedonia among groups, though the CUMS group showed a decreasing trend. This might be because CUMS affected sucrose consumption mainly in male (Dalla et al., 2005), while we selected female mice since the effect of CUMS was more obvious in female mice than in male mice (Ma et al., 2023). According to the results of the TST, FST, and OFT, CUMS treatment in this study successfully induced depression-like behavior in a mouse model. Moreover, our study demonstrated that intervention with different concentrations of probiotics improved despair and anxiety behaviors in CUMS model, especially in the TST (Figure 1).

The ACTH level in serum, which is an HPA axis-related hormone, increased in the CUMS-treated group. This result was similar to those of previous studies (Zhang et al., 2020; Qian et al., 2021), and intervention with probiotics reversed these increases. The CORT is a hormone increased by stress and plays a significant role in the pathophysiology of MDD (Alenko et al., 2020). However, we did not observe significant changes in serum CORT in this study. A previous study reported that the increase of CORT in mice subjected to the CUMS procedure for 36 days was much lower than that in mice subjected to stress for 18 days, indicating the presence of adaptation mechanisms to stress (Pałucha-Poniewiera et al., 2020). The non-significant CORT changes in our study might be partly explained by the adaption of mice to long-term CUMS. Furthermore, BDNF showed no significant difference among groups. BDNF and estrogen receptors are co-expressed in some neurons, and estrogen may act directly through its receptors to regulate BDNF expression (Wei et al., 2017). Our female mice may be in different estrous cycles so BDNF was affected by not only CUMS treatment but also estrogen. The level of 5-HT, an important neurotransmitter, decreased after CUMS treatment and was increased by probiotics treatment, which is consistent with the findings of other studies (Deng et al., 2021; Huang et al., 2022). Probiotic treatment also reversed the brain morphological changes associated with CUMS treatment (Figures 2E, F).

Transcriptome studies demonstrated that our CUMS model was established successfully. The downregulated genes in the CUMS group were mainly enriched in the Wnt signaling pathway and tight junctions (Figure 3E), which are strongly associated with the nervous system or depression. Many reports have shown that many antidepression treatments play roles in relieving depression-like behavior by activating the Wnt signaling pathway (Zhao et al., 2019; Jing et al., 2020). Gormanns et al. reported that the tight junction pathway was dysregulated in patients with anxiety (Gormanns et al., 2011). Moreover, the expression of tight junction proteins was decreased in chronic mild stress mice (Martín-Hernández et al., 2023). Furthermore, the upregulated genes in the CUMS group were enriched in the apelin signaling pathway, neuroactive ligand-receptor interaction, calcium signaling pathway, and thyroid hormone signaling pathway (Figure 3G). Gok Oguz et al. analyzed the apelin level in peritoneal dialysis (PD) patients with depression and anxiety and found that it was significantly greater than that in PD patients without depression and anxiety (Gok Oguz et al., 2016). A previous study also revealed DEGs related to neuroactive ligand-receptor interactions in a chronic restraint stress (CRS) rat model (Yoo et al., 2022). The calcium signaling pathway was also enriched in CUMS models and depression patients (Kao et al., 2012; Chen et al., 2023). The upregulated genes in both the LOW and HIGH groups were significantly enriched in the PI3K-Akt signaling pathway (Supplementary Figure S1), which is consistent with previous research (Zhang et al., 2021; Zhou et al., 2021). Chaihu Shugan San can alleviate the depression behavior by activation of the PI3K/Akt pathway in CUMS mice (Zhang et al., 2021). Xiaoyaosan treatment can exert antidepressant effects through the PI3K/Akt signaling pathway in CUMS rats (Zhou et al., 2021). Liu et al. confirmed that *C. butyricum* could alleviate the relative proteins expression of PI3K-Akt signaling pathway in vascular dementia mouse model (Liu et al., 2019). This may be a potential direction for our further research.

Next, we analyzed DEGs in the CUMS group that were reversed by different concentrations of probiotics (Tables 1, 2). *Sfrp5*, secreted frizzled-related protein 5, was downregulated in the CUMS group, which is consistent with the finding that *Sfrp5* were downregulated in CRS mice model (Wang J. et al., 2023). The middle concentration of the probiotics reversed the increase in the expression of *Sfrp5*. *Angpt2* is a factor of Angiogenic, which plays an important role in vessel protection, repair and reconstruction (Lv et al., 2022). The microvasculature is involved in the regulation of many brain processes and, when impaired, is susceptible to lacunar and hemorrhagic stroke, cognitive dysfunction, and depression (Van Sloten et al., 2020). Jiang et al. demonstrated that fear stress might downregulate the expression of *Angpt2*, and the downregulation of *Angpt2* expression leads to hippocampal microvascular remodeling and brain function damage in pregnant rats (Jiang et al., 2023). Our results showed that the expression of *Angpt2* in CUMS mice was also downregulated, and high-concentration probiotics treatment upregulated the expression of *Angpt2* in CUMS mice.

The results of the GO term enrichment analysis of the genes whose expression was reversed by probiotics were concentrated on cellular components, glutathione transferase activity, and sodium ion transport. Glutathione plays an important part in protecting the brain against oxidative stress (Gibson et al., 2012). Using magnetic resonance spectroscopy (^1^H-MRS), Duffy et al. demonstrated that depressive symptoms in the elderly were associated with increased glutathione in the anterior cingulate cortex (ACC) (Duffy et al., 2015). Unfortunately, the DEGs upregulated in the CUMS group and reversed by probiotics did not show a clear association with the nervous system or depression.

As the basis of the microbiota-gut-brain (MGB) axis, the gut microbiota provides an environment for probiotics to participate in gut-brain communication. Many studies have indicated an association between depression and the gut microbiota (Rathour et al., 2023). The ANOSIM and PerMANOVA results (Figure 4; Supplementary Table S2; Supplementary Table S3) revealed significant differences between the CUMS group and the CON group, and both the probiotics and Fluoxetine treatments significantly changed the microbiota structure of the CUMS group. Our results found that the abundance of *Bacteroides caecimuris* was reversed by MID, HIGH, and FLX treatment. A previous study indicated that *Bacteroides* abundance was significantly decreased in MDD patients (Jiang et al., 2015). After the investigation of potential functions in each group, we found that the pathway PWY-7377, which was decreased in the CUMS group, was significantly increased in LOW and HIGH compared with CUMS. PWY-7377, also known as cob(II)yrinate a,c-diamide biosynthesis I, is an anaerobic pathway for vitamin B12 biosynthesis. A lack of vitamin B can influence memory function, cognitive impairment and dementia, and vitamin B is closely connected with depression (Mikkelsen et al., 2016). Low vitamin B12 levels have been found in studies of depressive patients (Coppen and Bolander-Gouaille, 2005). Therefore, the PWY-7377 pathway is a noteworthy pathway related to depression.

According to the results of the correlation analysis between transcription and the gut microbiota, we found that *Tbxas1* was positively related to *B*. *caecimuris. Tbxas1* encodes the enzyme thromboxane A synthase 1 and participates in the arachidonic acid (AA) cascade, and decreased expression of *Tbxas1* caused an increase in the risk of depressive symptoms (Park et al., 2024).

In addition, *P*. *distasonis* was positively related to *Cldn2. Parabacteroides distasonis* is a type strain of *Parabacteroides.* The genus *Parabacteroides* commonly colonizes the gastrointestinal tract of many species (Ezeji et al., 2021). *Parabacteroides distasonis* was reported to alleviate the depression-like behavior of mice subjected to chronic restraint by increasing the 5-HT level in the hippocampus and inhibiting the kynurenine metabolic pathway (Deng et al., 2021). Moreover, Chaihu Shugan San was suggested to alleviate depression-like behavior in CUMS mice by increasing intestinal *P. distasonis* abundance and the levels of the bile acids hyocholic acid and 7-ketoDCA (Ma et al., 2022). In this study, we found *P. distasonis* significantly decreased in CUMS group and restored to the control level after probiotics intervention (Table 4). *Cldn2* encodes Claudin-2, a tight junctional protein present in the epithelial tight junctions of the choroid plexus forming the blood-CSF (cerebrospinal fluid) barrier (Wolburg et al., 2001). The decrease in *Cldn2* expression led to an increase in the permeability of the blood-CSF barrier and more severe neuroinflammation and an increased risk of depressive-like behavior (Wang W. et al., 2023). In our study, the expression of *Cldn2* decreased in the CUMS group and increased after probiotics intervention (Supplementary Table S7). Therefore, the probiotics may alleviate the depression-like behavior of CUMS mice by regulating the expression of *Cldn2* and further affecting tight junction proteins.

Therefore, we demonstrated that probiotics treatment may affect the expression levels of *Tbxas1* and *Cldn2* by changing the abundance of *B*. *caecimuris,* and *P. distasonis*.

In conclusion, we successfully established a CUMS-induced depression model, and probiotics significantly alleviated depression-like and anxiety behaviors in CUMS-induced mice. The effect of probiotics might be achieved by changing the expression of PFC genes and the abundance of gut bacteria that were associated with depression. The main limitation of our study is that the small sample size resulted in some results showing corresponding trends but no significant differences. To improve our study, we can further use both male and female mice and increase the sample size. In addition, transcriptome analysis revealed several pathways associated with probiotics treatment, but these pathways were not validated. Our next research direction could consider the PI3K-Akt signaling pathway and the Wnt signaling pathway as the midpoint for the probiotics mechanism of action.

## Data Availability

The RNAseq and gut microbiota data presented in the study are deposited in the GenBank Sequence Read Archive, accession number PRJNA1079201.
