# Supplementary figures and images for: Bacillus coagulans and Clostridium butyricum synergistically alleviate depression in a chronic unpredictable mild stress mouse model through altering gut microbiota and prefrontal cortex gene expression
Source: Front Pharmacol. 2024 May 23;15:1393874. doi: 10.3389/fphar.2024.1393874 (PMC11158626; doi:10.3389/fphar.2024.1393874)

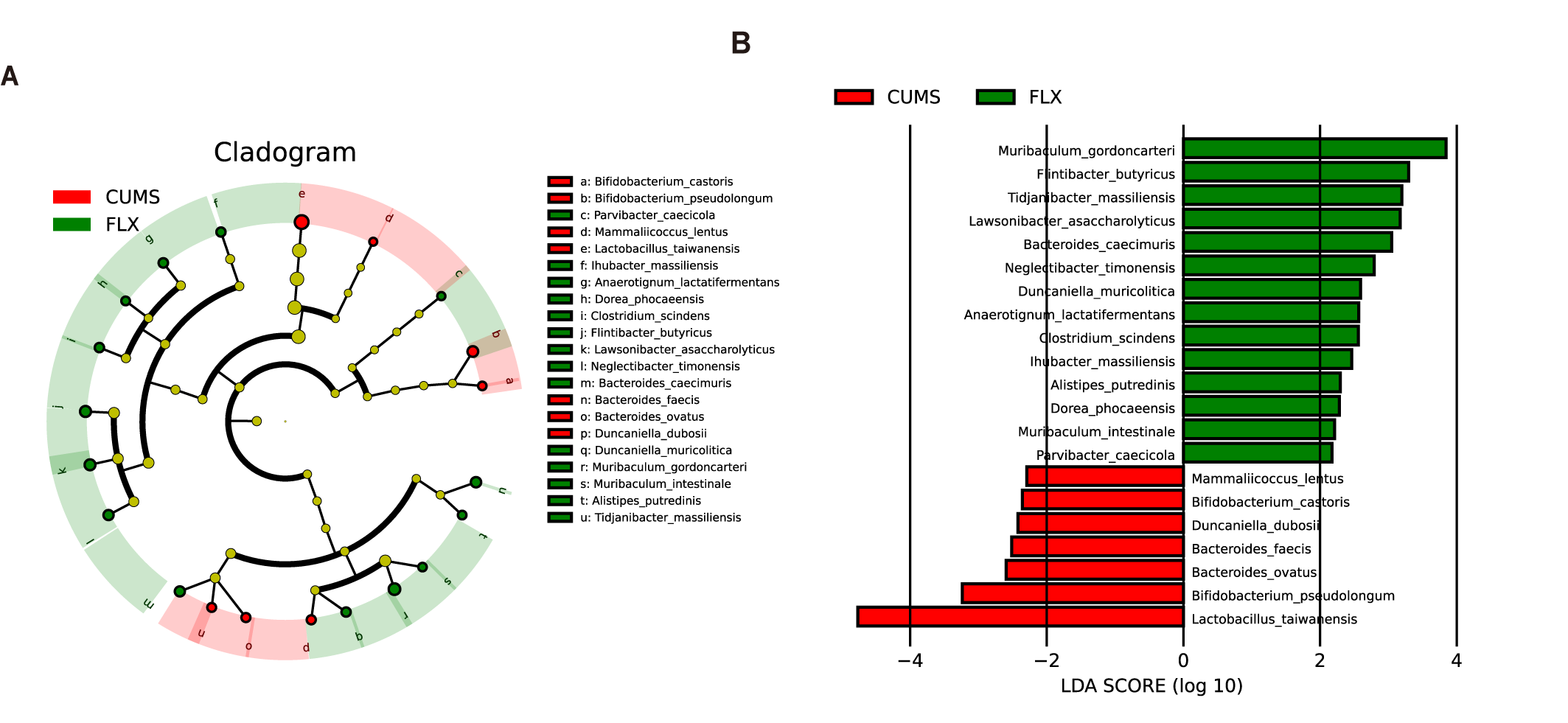

Supplement: Supplementary file 1 [file Image3.TIF]

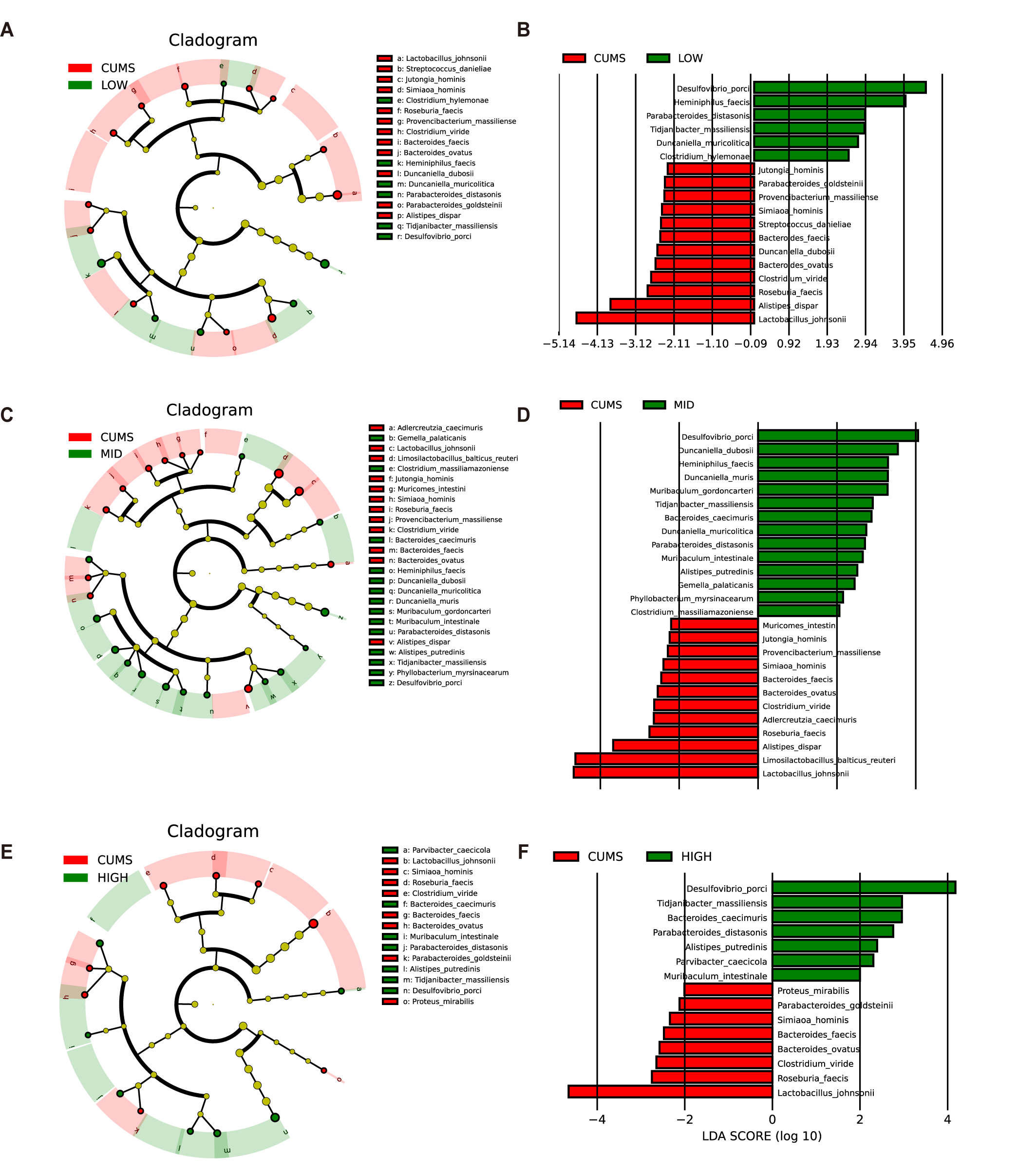

Supplement: Supplementary file 2 [file Image2.TIF]

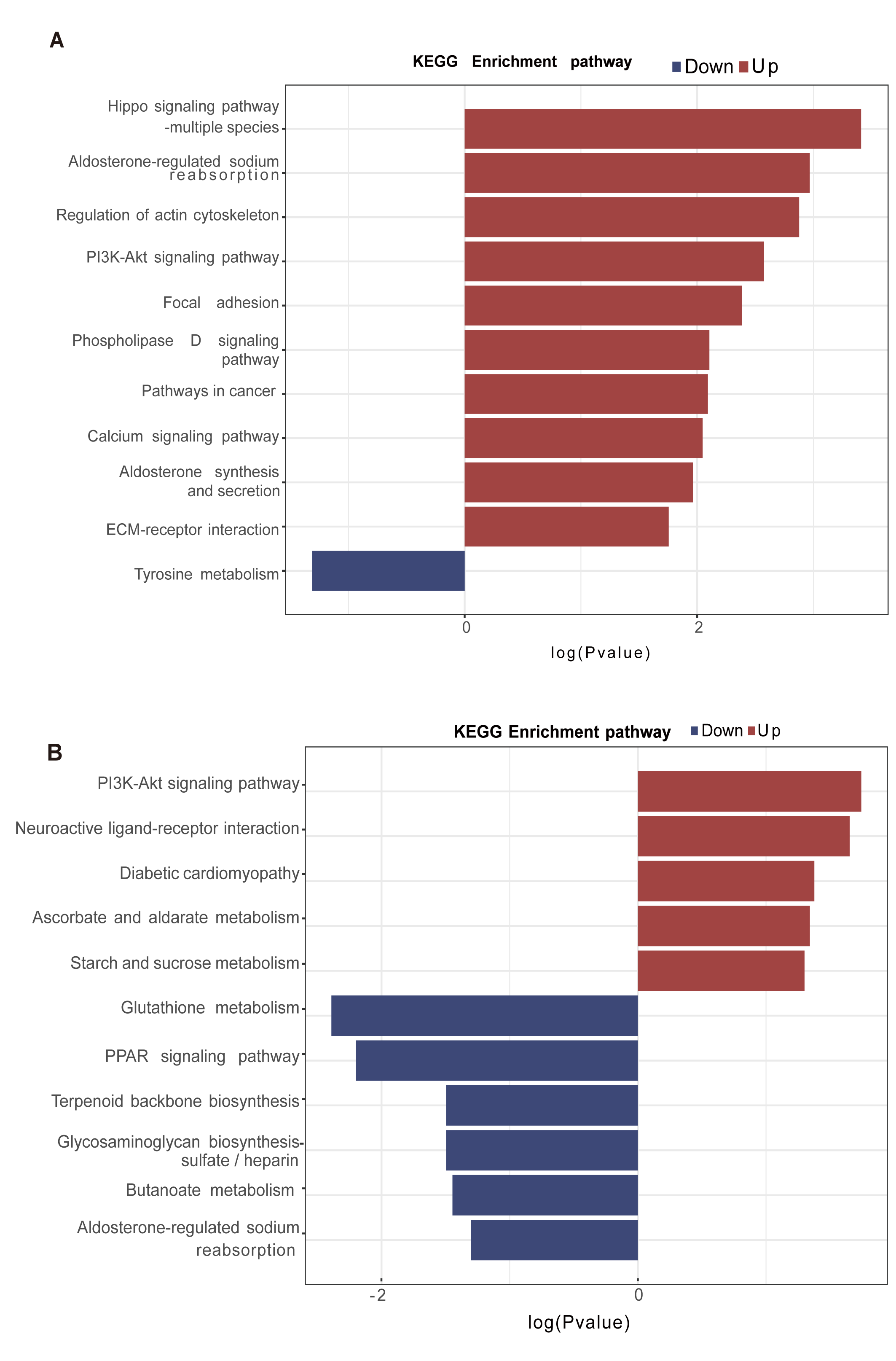

Supplement: Supplementary file 3 [file Image1.TIF]
